# From the real device to the digital twin: A coupled experimental-numerical strategy to investigate a novel bioresorbable vascular scaffold

**DOI:** 10.1371/journal.pone.0252788

**Published:** 2021-06-04

**Authors:** Luca Antonini, Francesca Berti, Benedetta Isella, Dipok Hossain, Lorenzo Mandelli, Giancarlo Pennati, Lorenza Petrini

**Affiliations:** 1 Laboratory of Biological Structure Mechanics, Department of Chemistry, Materials and Chemical Engineering “Giulio Natta”, Politecnico di Milano, Milano, Italy; 2 Department of Civil and Environmental Engineering, Politecnico di Milano, Milano, Italy; University of Vigo, SPAIN

## Abstract

The purpose of this work is to propose a workflow that couples experimental and computational activities aimed at developing a credible digital twin of a commercial coronary bioresorbable vascular scaffold when direct access to data about material mechanical properties is not possible. Such a situation is be faced when the manufacturer is not involved in the study, thus directly investigating the actual device is the only source of information available. The object of the work is the Fantom® Encore polymeric stent (REVA Medical) made of Tyrocore™. Four devices were purchased and used in mechanical tests that are easily reproducible in any mechanical laboratory, i.e. free expansion and uniaxial tension testing, the latter performed with protocols that emphasized the rate-dependent properties of the polymer. Given the complexity of the mechanical behaviour observed experimentally, it was chosen to use the Parallel Rehological Framework material model, already used in the literature to describe the behaviour of other polymers, such as PLLA. Calibration of the material model was based on simulations that replicate the tensile test performed on the device. Given the high number of material parameters, a plan of simulations was done to find the most suitable set, varying each parameter value in a feasible range and considering a single repetitive unit of the stent, neglecting residual stresses generated by crimping and expansion. This strategy resulted in a significant reduction of computational cost. The performance of the set of parameters thus identified was finally evaluated considering the whole delivery system, by comparing the experimental results with the data collected simulating free expansion and uniaxial tension testing. Moreover, radial force testing was numerically performed and compared with literature data. The obtained results demonstrated the effectiveness of the digital twin development pipeline, a path applicable to any commercial device whose geometric structure is based on repetitive units.

## 1 Introduction

The global burden of cardiovascular disease (CVD) is not only a health issue but an economic challenge to healthcare systems that is expected to grow exponentially in future years. CVD has major economic consequences that affect individuals, health systems, and societies across the globe. The World Heart Federation has estimated that by 2030, the total global cost of CVD is set to rise from approximately $ 863 billion in 2010 to a staggering $ 1044 billion [1]. Among CVDs, coronary artery disease causes over 4 million deaths per year. The common treatment consisted of the percutaneous transluminal angioplasty (PTA), which was then upgraded into the PTA followed by implantation of a stent. Over time, these medical devices experienced great technological innovation up to their establishment as the golden standard for obstructive atherosclerotic vascular disease [2, 3]. Initially, they were made of bare metal (BMS), then replaced by metal covered by a drug-eluting layer (DES) [4]. In 2016, the global coronary stents market was accounted for $ 5.4 billion and by 2023 is expected to reach $ 7.1 billion [5]. However, permanent devices were proved to cause long-term complications limiting the successful vascular repair, opening the community toward the use of bioresorbable vascular scaffolds (BVSs), which are tailored to temporarily sustain the injured coronary artery and then to be resorbed when the vessel has healed [6].

Numerical simulations are nowadays generally accepted as a useful tool during the device design phase, to integrate and enrich data provided by in-vitro tests. More recently, in-silico evidence has been accepted also by the regulatory authorities to support device marketing authorization, i.e. through modelling and simulation [7]. However, the potential of computational analyses is not limited to support manufacturers during the pre-commercial phase, but it can be effectively exploited by research centers or hospitals to run studies dedicated to investigating and verifying the safety and reliability of commercial devices. This is even more useful for BVS, due to the complexity of their behaviour related to time-dependent response. A device digital twin has to be able to replicate the actual functional behaviour in a virtual but realistic scenario. The current debate focuses on the definition of good practices for the preparation of reliable, accurate, and credible models [8–10].

Many examples of metallic stent models were used in the literature of coronary numerical simulations [11–13]: in the majority of the cases, the geometry was reconstructed from optical observation of the real device and the material properties taken from the literature of standard metals, such as cobalt-chromium and stainless steel. Focusing on the recent literature, few studies are reviewing the preparation of validated stent models [14, 15], always considering metallic devices, which involved experimental tests for validating the choice of numerical parameters. On the other hand, there is reduced literature regarding BVS characterization and modeling, mainly devoted to assessing the mechanical response of first-generation poly-L-lactic acid (PLLA) based scaffolds, such as Absorb® (Abbott Vascular, Santa Clara, California, USA) [6, 16, 17]. Moreover, this polymer is known to exhibit complex mechanical properties, such as visco-plasticity, temperature, and strain rate dependency: ad-hoc experiments are required to fully characterize this behaviour and most of the studies neglect part of the phenomenology. It is quite common to use an elastic-plastic material model as in [18–20], calibrated either using literature data or through material specimens: it requires few parameters as input (Young’s Modulus, Poisson’s ratio, and a plastic hardening coefficient), but it does not account for any viscosity or temperature/rate dependency. Wang et al. [6] used an elastic-visco-plastic Johnson-Cook model, calibrating the parameters over a few experiments on material specimens: this constitutive model can account for strain rate and temperature dependency. In the studies of Eswaran et al. and Debusschere et al. [21, 22] an accurate material model, sensitive to all the aforementioned dependencies, was implemented through a user subroutine starting from the Bergström and Boyce model [23] and calibrated over a wider range of tests. Dreher et al. [24] employed the Advanced Flow Evolution Network model to be able to capture not only the viscous behaviour but also the post-yield softening of PLLA. Finally, Bobel et al. [25] used the Parallel Rheological Framework (PRF) which is a model used for polymers and elastomers that exhibit a non-linear viscous behaviour and undergo large deformations [26].

In this context, the present work aims to define a pathway useful for whoever is interested in performing comparative in-silico preclinical studies of bioresorbable cardiovascular stents, independently from the manufacturer. In this situation it is reasonable to assume that CAD drawings, as well as material data or samples for material mechanical characterization, are not provided by the producer company. With this perspective, this study proposes an experimental and computational strategy that allows developing a digital twin of a commercial BVS, exploiting and maximizing the information available from few simple in-vitro tests on a small number of purchased devices.

In particular, the occasion for approaching and developing this theme was given by the European project InSilc, devoted to the set-up of an in-silico platform for the prediction of coronary stents implantation performance in the individual cardiovascular physiology. It was decided to study the novel Fantom® Encore Sirolimus-Eluting Bioresorbable Coronary Scaffold without the involvement of the manufacturing company (REVA Medical Inc., San Diego, CA, USA). This BVS is a third-generation coronary stent, differing from previous generations due to the proprietary tyrosine-derived polymer (Tyrocore™), specifically designed for vascular scaffold applications. Some improvements were introduced to achieve broader adoption: thinner struts, full radiopacity, no discernible coating layer, and ease-of-use. Despite these advantages, to the best of the authors’ knowledge, the information about the stent performances is very poor and no data are available in the literature about the Tyrocore™ material properties and the stent mechanical response. Hence, some delivery systems were purchased and ad hoc experimental tests were performed on them to know the material phenomenology, develop the device digital twin, and checking its reliability with respect to the goal of InSilc.

## 2 Materials and methods

**Fig 1:**
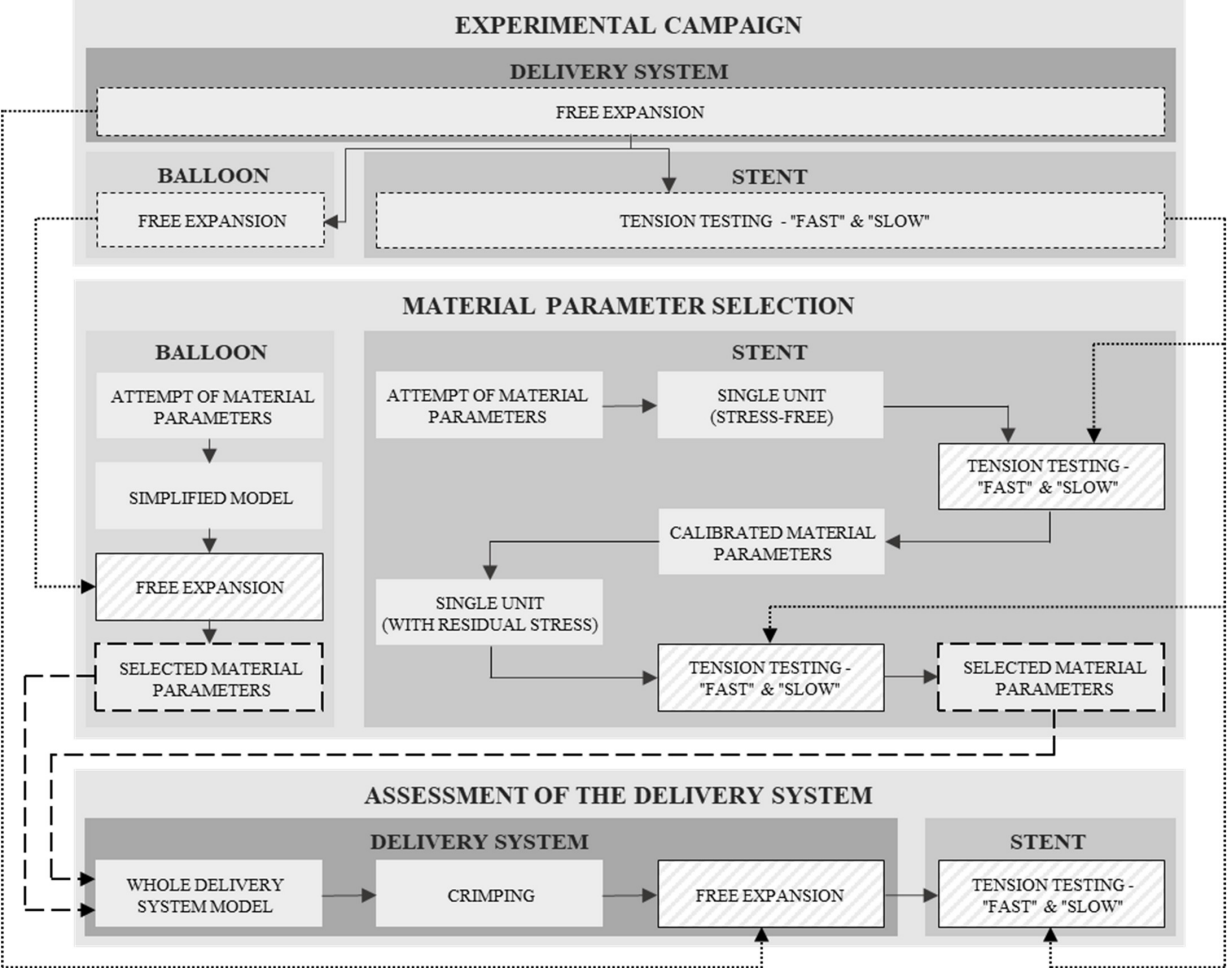
Schematic representation of the workflow of the study.

In this paper, a workflow that couples experimental and computational activities aimed at developing a credible digital twin of the Fantom® Encore polymeric stent (REVA Medical) using only information available from purchased delivery systems, is presented. The BVS is obtained by a laser cutting process on Tyrocore^TM^ tube previously formed to the desired diameter (for a polymeric device, it corresponds to the nominal diameter of the expanded stent) and coated with a mixture of Tyrocore^TM^ and Sirolimus. It is then crimped together with a semi-compliant balloon mounted on the catheter to get a complete delivery system, ready for clinical application. The study workflow is described in Fig 1. The activity was organized in successive phases. The first of these was the experimental campaign, which aimed to collect all the possible information on the mechanical behaviour of the available delivery system samples (composed of the stent crimped on the balloon). Initially, free expansions of delivery systems were performed, and then individual balloons and stents were tested. In particular, the balloons were deflated and newly free expanded, while the stents, in the expanded configuration, were subjected to tension testing. The collected data on balloons and stents were used in the second phase aimed at the generation of the finite element (FE) model of the devices. A crucial step in the development of the models is the selection of the material model and hence the calibration of the material parameters. In this work, as better described in the following, a simple linear elastic model was adopted for the balloon [27], which allowed to use a straightforward procedure based on experimental results for the parameter calibrations. Concerning the stent, the experimental results confirmed the rate-dependent mechanical behaviour of Tyrocore^TM^, requiring the adoption of a non-linear viscous model, characterized by several parameters. Accordingly, it was necessary to develop a specific strategy for material parameter calibration capable of making the most of the only information available from the force-displacement curves obtained from the uniaxial tension testing. In particular, a repetitive unit was recognized into the stent design and the FE model of a single repetitive unit was used for the simulations. At first, the residual stresses due to the previous loading history of the stent (which before the tension testing underwent crimping and free expansion) were neglected and the unit in the stress-free expanded configuration was considered. The tension testing was numerically reproduced on the unit, investigating a very large set of parameters. The results allowed identifying the set of parameters better fitting the experimental data. In the second phase, the set choice was verified, repeating the simulation of tension testing using the identified set on the unit that, after simulations of crimping and free-expansion, was in the expanded configuration with residual internal stresses. This strategy allowed performing a high number of simulations while keeping the computational costs low. Finally, performance assessment of the digital twins, thus obtained, was carried out by subjecting, first, the delivery system to free expansion and, then, the expanded stent to uniaxial longitudinal tension testing, and comparing the numerical results with the experimental ones.

### 2.1 Experimental campaign

The experimental campaign was performed using four coronary BVSs, appositely purchased (according to the budget of the project), and consisted of free expansion tests on the delivery systems and uniaxial tension testing on the stents in the post-expansion configuration. All the experimental tests were conducted in the water at 37 ± 1°C. All the simulations were performed using the commercial code Abaqus 2018 (Simulia Corp. USA).

#### 2.1.1 Stent free expansion

**Fig 2:**
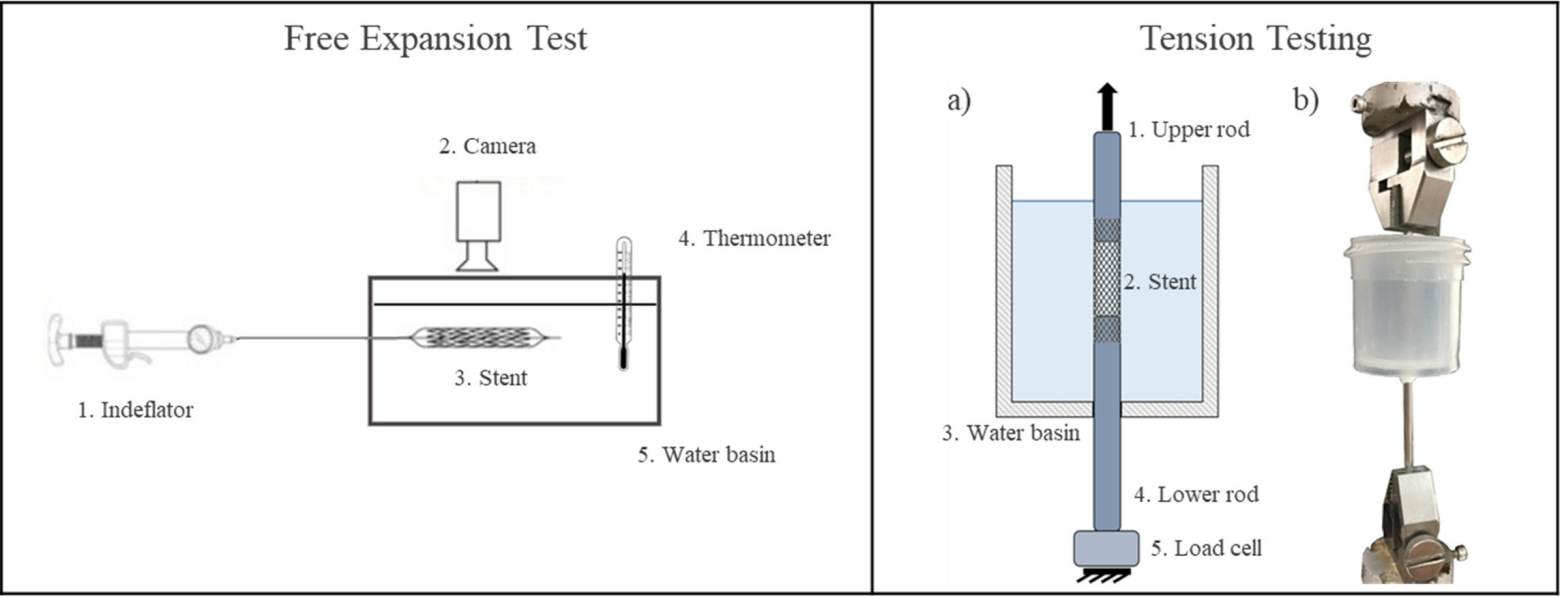
On the left: Schematic representation of the experimental set-up for the free expansion tests. On the right: experimental set-up for the uniaxial tension testing: a) schematic representation, b) image of the actual set-up).

Free expansion tests were performed following the protocol suggested by the device instructions for use to obtain the pressure-diameter curves. The protocol consisted of three phases: expansion, holding, and release. In particular, 30 seconds holding phase, during which the pressure of the balloon was kept constant to allow the creep phenomenon to occur. The set-up for free expansions comprised (Fig 2, on the left): an indeflator (MEDFLATOR, Inflation Syringe System, MedexTM); a thermometer, and a high-resolution camera (Canon EOS 6D) equipped with a macro-objective (Canon MP-E 65mm f/2.8 1-5x). The indeflator was directly connected to the delivery system and the operator infused water in the system by turning the screw of the syringe up to a maximum pressure of 7 atm in about 10 s. After this step, the screw was maintained fixed and then rotated in opposite direction to manage the holding phase and the release phase. The stent-balloon system was fixed inside a water container at 37 ± 1°C. The infusion medium used for these experiments was distilled water mixed with blue alimentary dye, which allowed to distinguish the presence of liquid inside the catheter and balloon.

During the whole experiment, the operator maneuvering the indeflator declared with his/her voice the progressive change in the pressure value that was reached. In this way, during the post-processing phase, it was possible to select those video frames corresponding to the pressure of interest, namely at each atmosphere. Subsequently, each frame was analyzed using ImageJ software (National Institute of Health, USA) to evaluate the measures of proximal, central, and distal diameters at targeted pressure values. To account for inter/intra-user variability, the diameter values were obtained as an average of six values (3+3) taken independently by two operators. Data variability was accounted for as the range between minimum and maximum values. One well-known phenomenon during free expansion tests is the “dogboning”, which consists of the anticipated opening of the proximal and distal segments. During this phase, the opening of the stent occurs dynamically and, consequently, there is great variability in the measurement of diameters [16]. The dogboning phase is considered completed when there is no longer a significant difference between the diameters of the stent ends with respect to the central one. At this final stage, the fully expanded stent is representative of its configuration when deployed into the artery: for this reason, great accuracy is required by the model to represent this configuration. In this work, with "post-dogboning configuration" will be indicated the configuration reached by the stent at the end of the phase of the dogboning phenomenon, while with "final configuration" the state reached once the pressure is equal to the nominal value of 7 atm.

#### 2.1.2 Stent tension testing

The experiments were performed using BOSE Enduratec ElectroForce® 3200 on which a 22N load cell was mounted. Two small cylindrical metal rods, with a diameter that fits the inner diameter of the stents, were inserted and glued at two rings at the ends of the free-expanded device. A water basin was mounted onto the bottom metal rod and fixed using Teflon tape (Fig 2, on the right) to perform the tests in the water at 37 ± 1°C. Two different protocols (“fast” and “slow”), each one involving two devices, were defined for the displacement controlled tension testing to evaluate the visco-elastic and visco-plastic response expected from this unknown polymeric material. In the first case, the “fast” one, the test consisted of an axial displacement up to 3 mm in 30 s, followed by 30 s of holding and 30 s of release. In the other case, the “slow” one, the 3 mm displacement was applied in 120 s, followed by holding and release steps each lasted 120 s. Load-displacement curves were evaluated.

#### 2.1.3 Balloon free expansion

The four deflated balloons were tested again after free expansion experiments. Using the same expansion protocol previously adopted for the delivery system, it was possible to test the mechanical properties of the balloon up to a pressure of 7 atm and to consider variability among different samples.

### 2.2 Material models

#### 2.2.1 Stent material model

In order to succeed in reproducing all the mechanical characteristics of a polymer, the chosen material model was the Parallel Rheological Framework (PRF), which had already been used to describe PLLA [25]. Indeed, this model is suitable for materials that exhibit non-linear viscous behaviour and undergo large deformations [26]; it has also the advantage of being directly implemented in Abaqus. It consists of different parallel branches among which at most one can be elastic-plastic while the others are visco-elastic. For the sake of simplicity and to reduce the number of parameters to identify in the calibration process, a single visco-elastic branch parallel to an elastic-plastic one was considered. Specifically, all the elastic elements are described with the same hyper-elastic law whose stiffness is split into the different branches on the basis of a parameter known as stiffness ratio (SR), while dashpots are modeled with a creep law. SR parameter can vary between 0 and 1. In the case of this study, since only two branches are considered, when SR tends to 0 the contribution of the viscous branch is minimized, while a high value of SR, close to 1, means the dominance of the viscous branch over the elastic-plastic branch.

A Neo-Hooke hyper-elastic law (Eq 1) was used for the springs and a power-law strain hardening model (Eq 2) for the creep law:

W=C10(I¯1−3)+1D1(Jel−1)2 (Eq 1)

$${\dot{\bar{\varepsilon }}}^{cr}={\left(A{\tilde{q}}^{n}{\left[(m+1){\bar{\varepsilon }}^{cr}\right]}^{m}\right)}^{\frac{1}{m+1}}$$ (Eq 2)

where ${\bar{I}}_{1}$ is the first deviatoric strain invariant, *J*^*el*^ is the Jacobian, and *C*_10_ and *D*_1_ are temperature-dependent material parameters. In Eq 2, ${\bar{\varepsilon }}^{cr}$and ${\dot{\bar{\varepsilon }}}^{cr}$ are the equivalent creep strain and strain rate, $\tilde{q}$ is the von Mises equivalent stress, and *A*,*m* and *n* are material parameters.

Finally, plasticity was defined with a power law (Eq 3) to predict a visco-plastic behaviour with ideal plasticity (the increase in plastic strain does not lead to an increase in the value of the stress that is kept constant):

$$\sigma_{y}=\left[1+{\left(\frac{{\dot{\varepsilon }}_{pl}}{mult}\right)}^{\frac{1}{e}}\right]\sigma_{y0}$$ (Eq 3)

where *σ*_*y*_ is the yield stress, ${\dot{\varepsilon }}_{pl}$ is the equivalent plastic strain rate, *σ*_*y*0_ is the static yield stress, *mult* and *e* are material parameters.

In these conditions it was necessary to calibrate nine parameters: *C*_10_, *D*_1_ for the hyper-elastic response, *SR* to split the stiffness into the two different branches, *A*,*n* and *m* for the visco-elastic dashpot and *mult*, *e* and *σ*_*y*0_ for the plastic response.

#### 2.2.2 Balloon material model

A linear elastic model was adopted for describing the balloon elastomeric material behaviour [27].

### 2.3 Development of the finite element models

#### 2.3.1 REVA Fantom^®^ Encore

**Fig 3:**
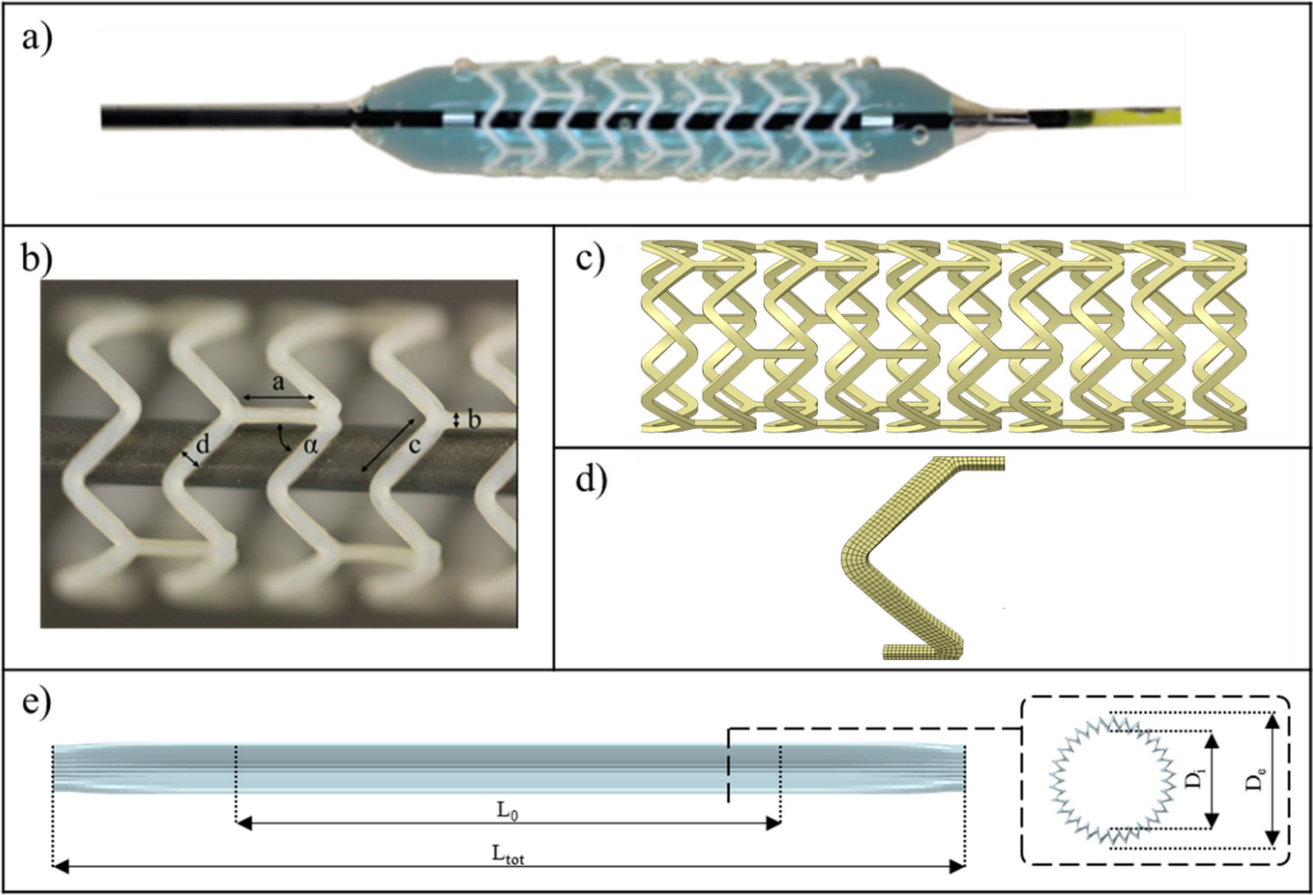
a) Delivery system in its full-expanded configuration; b) geometrical parameters of interest extracted through the software ImageJ; c) stent reconstructed geometry d) meshed V-shaped; e) geometry of the multi-wings structure of the balloon.

The geometrical model of 3.5 x 12 mm REVA Fantom^®^ Encore was obtained from photographic images of the devices (Fig 3A) in the post-expanded configuration. The first step of the geometrical reconstruction consisted of the extraction of the parameters of interest (Fig 3B) from the optical images through the software ImageJ (National Institutes of Health, Bethesda, Maryland, USA). Each measure was taken three times by two different operators in order to address problems of inter-operator and intra-operator variability. After the extraction of data, the geometry was reconstructed in SolidWorks (Dassault Systèmes, Vélizy-Villacoublay, France). Given the symmetries present in the structure, a V-shaped unit was realized and repeated to obtain the entire stent. The geometry was then wrapped around a cylinder and extruded in order to obtain the right wall thickness (Fig 3C). Then the geometry was discretized (Fig 3D) using HyperMesh (Altair HyperWorks, 2017). The mesh consisted of hexahedral elements with reduced integration (C3D8R), particularly suitable to describe simulations in which bending assumes specific importance and to implement PRF. The dimension of elements was also carefully analyzed and after a mesh sensitivity study based on both crimping and tension testing performed on a single V-shaped unit, a mesh with three elements in the thickness was chosen corresponding to 1071 elements for the V-shaped unit (and a total of 63144 for the whole stent).

#### 2.3.2 Balloon

The balloon folding was modeled in a simplified manner [27, 28] to properly mimic its pressure-diameter relation and allow the correct expansion of the stent. This was obtained by considering the presence of thirty wings along the balloon surface, rather than describing the three major folds of the real balloons, as suggested by Martin et al. [29]. An illustration of the balloon with its tapered extremities is provided in Fig 3E. The geometrical dimensions relative to the total length of the balloon (*L*_*tot*_) and to the length of the central portion (*L*_0_) have been chosen by observing the real device, while the diameter value (*D*_0_) has been dictated by the need to have a balloon that at zero pressure can be contained in the lumen of the crimped stent, Moreover, *D*_0_ is related to *D*_*i*_ and to the number of folds to generate a model with the same expanded diameter of the actual balloon.

The geometry was discretized using HyperMesh, realizing a final mesh of 6480 quadrilateral membrane elements (M3D4). The thickness of the balloon, equal to 0.02875 mm, was measured with a micrometer and assigned to the model.

### 2.4 Material parameters selection

For the selection of the material model parameters, some of the experimental tests were simulated: starting from attempt values of the parameters, they were suitably modified up to obtain a good fitting between numerical and in vitro results.

#### 2.4.1 Stent material parameters

**Fig 4:**
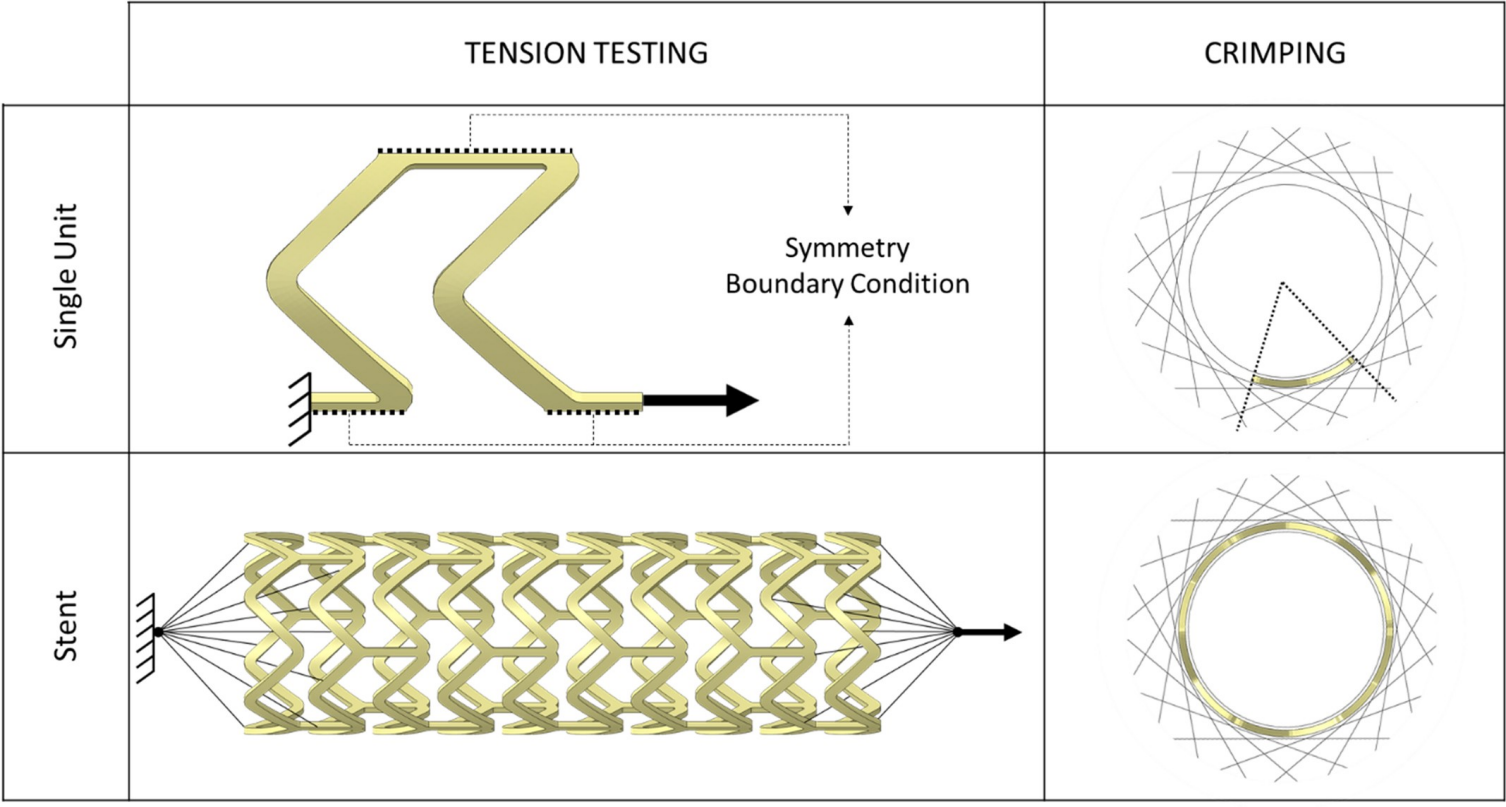
Model of single unit extracted from the whole stent with boundary conditions for tensile and crimping test simulations. The unit corresponds to a fifth of the entire stent length and a sixth of the circumference (upper part). Model of the whole stent with boundary condition for tensile and crimping test simulations (upper part) comparison between the boundary conditions applied to the whole stent and the single unit initial for tension testing simulations.

Considering the high number of parameters required by PRF model and the limited number of experimental tests available, it was decided to perform a set of simulations in which the material parameter values were variated in a feasible range. As already introduced, aiming to reduce the computational costs, in the prevision of the great number of simulations that had to be performed, a repetitive unit was identified thanks to the symmetries of the stent design. This single unit corresponds to a sixth of a double ring in the circumferential direction and to a thirtieth of the entire stent (Fig 4 –upper part). Through preliminary simulations, it was verified that the single unit was representative of total stent behaviour: in particular, a cylindrical reference system was created coaxial with the stent longitudinal axis, circumferential symmetry conditions were applied on the circumferential cuts, one longitudinal extremity was fixed while the opposite one loaded with a scaled displacement (Fig 4). Such a single unit was used to simulate the in-vitro uniaxial tension testing, considering fast and slow protocols. Suitable boundary conditions were imposed to reflect the behaviour of the whole stent (Fig 4, upper part). The simulation was set to reach a displacement equal to 30% of the gauge length (3 mm for the whole stent corresponds to 1 mm for the single unit) in 30 s. Abaqus/Standard was used to run the analyses and the obtained numerical load-displacement curves were then compared with the experimental ones.

The values of the nine material parameters were initially selected thanks to manual tuning, starting from literature data [30]. However, considering the difficulty of manually calibrating a model with such a large number of parameters and in order to optimize the results in terms of mechanical description, a further automatic tuning process was performed. An interval of values, centered on those obtained from the manual process, was defined; from it, three different values were selected. Finally, combining these values, 3^9^ = 19683 material models were generated and associated with the unit to simulate the tension testing. The outcome of each simulation, in terms of the force-displacement curve, was compared to the experimental data. In particular, it was chosen to identify the most accurate set of parameters as the one minimizing the overall differences between the numerical and experimental force, acquired at defined displacements.

Up to this point, the simulations were run on the single unit of the stent in the stress-free expanded configuration. In the reality, the processes of crimping and free expansion performed before tension testing generated stresses into the stent. As these residual stresses may have a relevant impact on the tensile response of the material, further analyses were performed including the simulation of crimping and free expansion. The crimping was carried out using 18 rigid planes [31], circumferentially arranged around the unit, and imposing a radial displacement so to reach a final external diameter of 1.5 mm after 30 s, followed by other two phases, namely holding and release of the duration of 60 s and 30 s respectively. An internal support cylinder that reduces gradually its diameter was used in the simulation to avoid stent introflections. Such crimping protocol was arbitrarily chosen by the authors of this work since the procedure used by the manufacturer is unknown. The free expansion process was simplified using a cylinder instead of a balloon. The expansion was achieved by imposing a radial displacement to the cylinder to replicate the movement of the balloon in the experimental free expansions. Finally, the free-expanded unit was subjected to the tensile experimental protocols to account for the modifications obtained in the response due to residual stresses.

#### 2.4.2 Balloon material parameters

Computational simulations mimicking the expansion tests of balloons were set up in Abaqus/Explicit to find suitable values for Young’s modulus based on the obtained pressure-diameter curves. Poisson’s coefficient and density were imposed according to [32].

### 2.5 Assessment of the delivery system digital twin

In this phase, the whole stent was considered. It allowed appreciating the phenomena that require a full-length approach and possible aspects that were neglected during the material parameter selection where the single unit was preferred. Moreover, the production process was numerically reproduced: starting from the stress-free laser-cut stent geometry, the crimping of the stent on the balloon was simulated to obtain a realistic model of the delivery system (including residual stress in the stent). The performance assessment process was defined on the basis of the available experimental and literature data [33]. In particular, three tests were considered: free-expansion of the delivery system, tension, and radial force testing of the fully expanded stent.

#### 2.5.1 Stent free expansion

The first step of the assessment process consisted of a crimping and expansion simulation using Abaqus/Explicit. The crimping step was performed following the same protocol described in section 2.4.1 and applied to the device to take into account the residual stresses. The minimum diameter was set at 1.3 mm to gain a realistic post-crimping stent diameter.

After the crimping step, the balloon was expanded by applying in 10 s an increasing uniform pressure up to 7 atm (the same as the experimental procedure) to the inner surface of the balloon. According to in vitro tests, a successive 30 s holding phase was added to permit the creep phenomenon. The balloon was then deflated by applying a decreasing pressure reaching finally a slightly negative value. Contacts were activated to account for the interactions between the different parts using the General Contact method. Given the high variability of experimental results in the dogboning phase, in agreement with the literature [16], the pressure-diameter curves obtained computationally were compared with the average curves of experimental results at the end of the dogboning phase (“post-dogboning configuration”) and when the pressure reaches the nominal value of 7 atm (“final configuration”). Moreover, the numerical results were compared with the experimental measurements also in terms of internal diameter reached at the end of the pressure maintenance phase and external diameter of the fully-expanded stent (at the end of the balloon deflation phase).

#### 2.5.2 Stent tension testing

The second step of the assessment path included the simulation of the tension testing of the stent. A process of import from Abaqus/Explicit to Abaqus/Standard allowed considering the residual stresses and deformed configuration deriving from the previous steps.

The force-displacement curves obtained computationally with the “fast” and “slow” protocols and the average curves of the corresponding experimental results were compared considering the maximum force, reached during the loading phase, the force measured at the end of the relaxation phase, and the residual plasticity, namely the displacement at which a zero force is measured during the unloading phase.

#### 2.5.3 Stent radial force testing

A further computational test, to be compared no longer with experimental results carried out in this work (only a limited number of samples were available), but with performance declared by the manufacturer, was carried out. In the datasheet of the REVA Fantom^®^ Encore, the manufacturer states that its device is characterized by a radial strength of 0.22 N/mm. According to the standard guideline for this procedure [34], the radial strength is measured by subjecting the device to a recrimping test. The stent is placed inside a machine that, through the radial movement of rigid planes, progressively reduces the diameter of the stent. The test is characterized by a phase of diameter reduction followed by one in which the planes are moved radially in the opposite direction to allow the elastic recoil of the stent. The radial strength value is estimated based on what is observed in a diameter-radial force plot. In this curve, during the loading step, it is possible to identify an initial phase with a nearly constant slope that corresponds to the elastic response of the stent to the diameter reduction, followed by a phase in which the curve flattens due to the increase of plastic deformations. During the unloading step, the curve has again a roughly constant slope, whose entity has to be used in the estimation of the radial strength. The radial strength corresponds to the intersection value between the recrimping loading curve and a straight line parallel to the unloading curve, with an offset with respect to the starting point of the load curve itself that corresponds to a 15% compression [33].

This test was reproduced computationally in Abaqus/Explicit: the stent, after crimping and free-expansion, was subjected to recrimping up to reach a minimum outer diameter of 1.5 mm, using the same strategy described in section 2.4.1., without interposing the holding step between the crimping step and the release step.

## 3 Results

### 3.1 Experimental results

#### 3.1.1 Stent free expansion

The results of the free expansion tests in terms of pressure–inner diameter curves are shown in Fig 7. The diameter values are reported for the proximal, central, and distal regions of the devices. The average diameter value of the four stents was plotted and the variability was accounted for by error bars indicating the maximum and minimum value of diameter reached with respect to the mean value at the corresponding pressure.

#### 3.1.2 Stent tension testing

**Fig 5:**
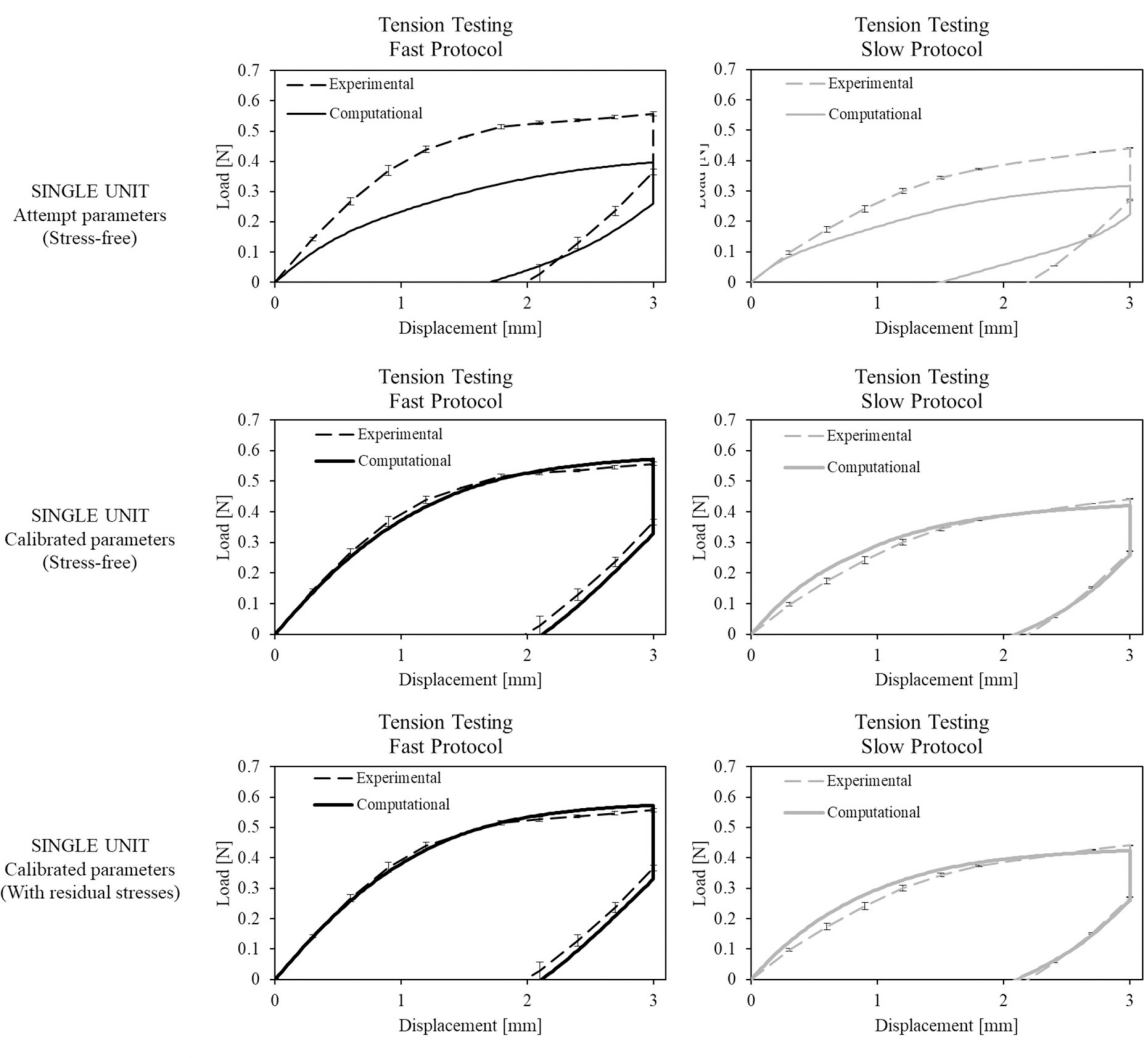
Comparison between experimental data of tension testing with “fast” and “slow” protocols and computational results obtained: i) at the beginning (upper part) and ii) at the end (central part) of the material parameter tuning process, using a stress-free single unit model; iii) at the end of the material parameter tuning process (lower part), using the single unit with residual stress. Error bars in the experimental curves indicate the maximum and minimum experimental value with respect to the mean value at the corresponding displacement.

The results in terms of load-displacement curves are reported for the two stents tested according to the “fast” tension testing protocol (Fig 5, left column) and for the two stents tested according to the “slow” tension testing protocol (Fig 5, right column). The error bars indicate the maximum and minimum experimental value with respect to the mean value at the corresponding displacement. The collected data show good repeatability despite the possibility of dedicating only two samples to each traction protocol.

#### 3.1.3 Balloon free expansion

**Fig 6:**
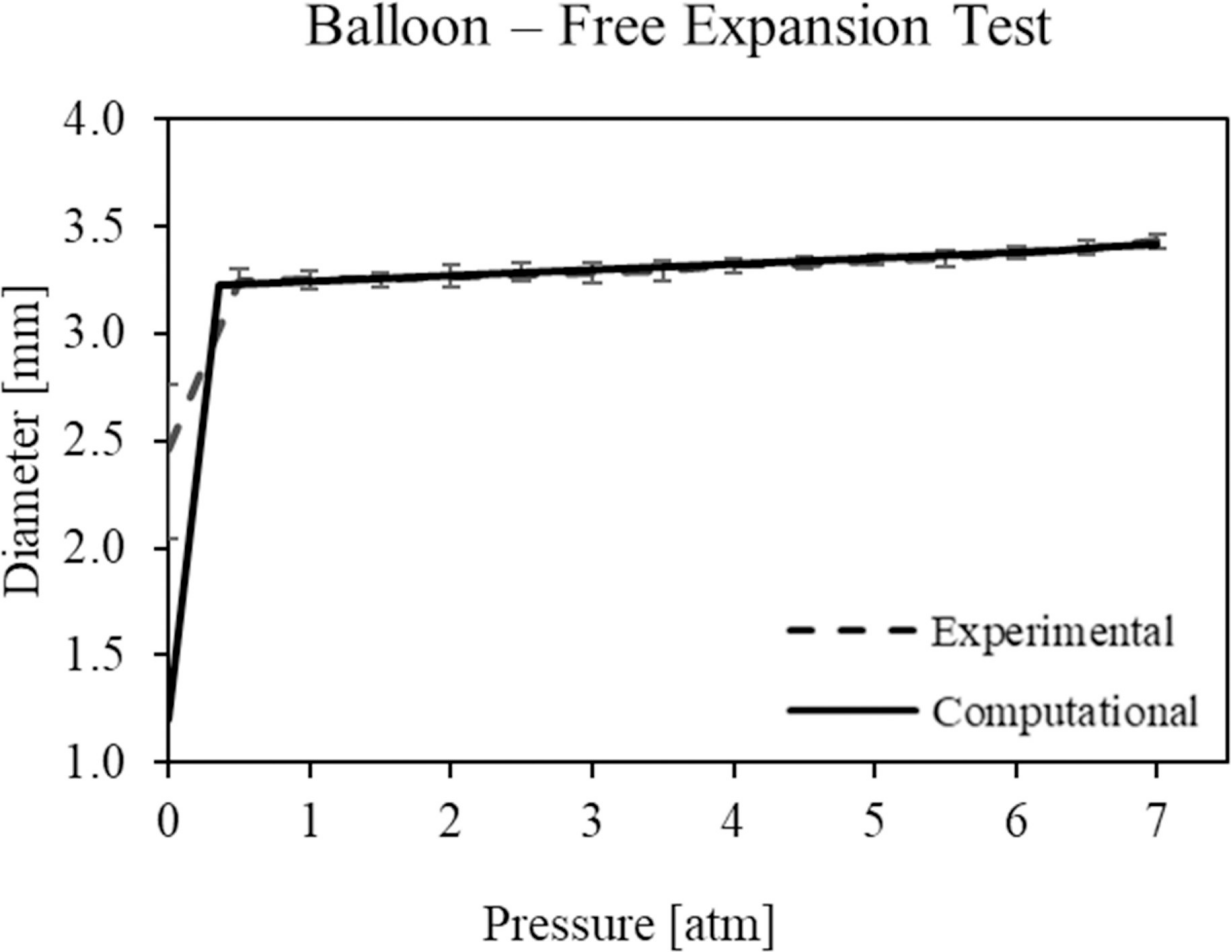
Comparison between the experimental and computational pressure-diameter curves from free-expansion of the balloons. Error bars indicate the maximum and minimum experimental value of diameter reached with respect to the mean value at the corresponding pressure.

A pressure-diameter curve was extracted from the free-expansions of the balloons (Fig 6). In particular, the first phase (up to 1 atm) indicates the unfolding of the balloon, while the second part of the curve represents gradual inflation.

### 3.2 Material parameters selection

#### 3.2.1 Stent material parameters

The simulation results of the tension testing with “fast” and “slow” protocols, performed on the stress-free single unit for identifying material parameters, are reported in Fig 5 (upper part). The load-displacement curves obtained using the set of parameters initially selected by manual tuning and the final set obtained from the automatic tuning process were compared with the experimental ones. The final set allowed having a good match. The errors obtained from comparing computational results of the final set with experimental values in terms of maximum force measured at the end of the loading phase, the force measured after the relaxation phase, and residual plasticity are reported in Table 1 –line 1. This set of parameters were also used to simulate the crimping of the stress-free single unit, its expansion, and then the tension testing, to include the effects of the residual stress on the computational outputs. As shown in Fig 5 (bottom), the match of the experimental and numerical load-displacement curves is still good, suggesting that the residual stresses don’t affect significantly the tensile behaviour and hence it is possible to adopt the final set of parameters (reported in Table 2) also for the future analyses. The error between the computational and the experimental data are reported in Table 1 –line 2.

**Table 1:** Percentage errors obtained comparing the experimental data of tension testing with “fast” and “slow” protocols and computational results got at the end of the material tuning process, using a stress-free stent single unit model (line 1) and a single-unit with residual stresses (line 2).

Errors [%]	Maximum Force		Relaxation Force		Residual Plasticity	
	“Fast”	“Slow”	“Fast”	“Slow”	“Fast”	“Slow”
Without residual stress	2.6	4.8	10.0	4.2	6.4	4.4
With residual stress	2.8	4.0	9.5	3.7	6.0	4.4

**Table 2:** Values of the parameters of the best material found with the calibration process.

*C*_*10*_ [MPa]	*D*_*1*_ [MPa^-1^]	*SR* [-]	*A* [(MPa s)^-1^]	*N* [-]	m [-]	*mult* [-]	*e* [-]	*σ*_*y0*_ [MPa]
340	0.0003	0.7	1E-06	2.0	-0.0001	3E-04	4.6	14.0

#### 3.2.2 Balloon material parameters

The material parameters selected for the balloon are summarized in Table 3. In Fig 6, the numerical results in terms of diameter vs pressure, obtained with the calibrated material, are compared with the experimental data.

**Table 3:** Parameters used to describe the elastomeric material of the balloon.

	Values
Density, ρ [ton/mm^3^]	1.0E-09
Elastic Modulus, *E* [MPa]	600
Poisson’s coefficient, ν [–]	0.45

### 3.3 Assessment of the delivery system digital twin

#### 3.3.1 Stent free expansion

**Fig 7:**
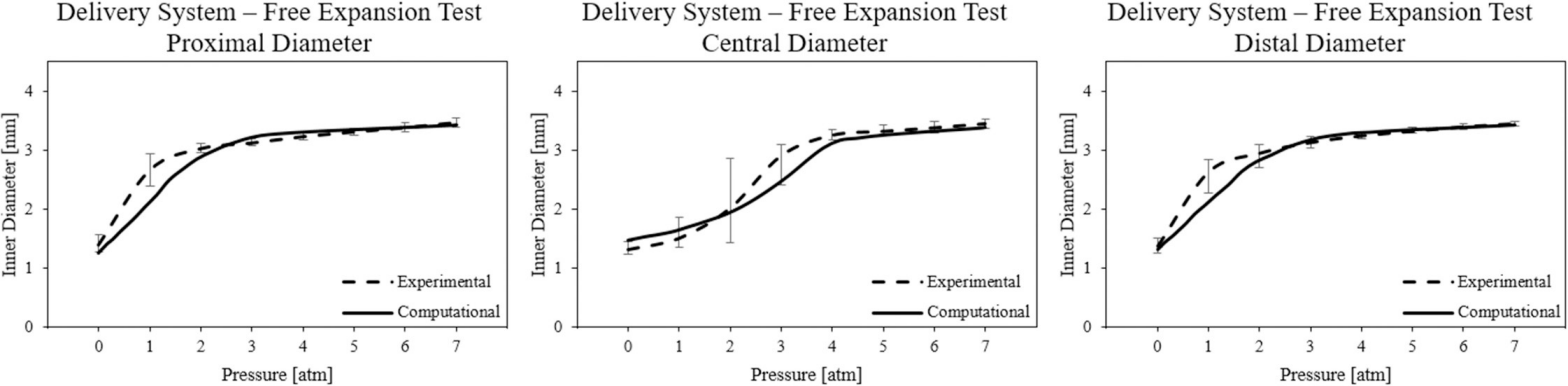
Pressure-diameter curves obtained experimentally and computationally from the free expansion of the delivery system. Error bars indicate the maximum and minimum experimental value of diameter reached with respect to the mean value at the corresponding pressure.

In Fig 7 the pressure-diameter curves obtained computationally are compared with the experimental ones. In particular, to assess the model credibility, the percentage errors with respect to the diameter values reached by the stent in the “post-dogboning” configuration (i.e. when the central diameter is not significantly different from those measured at the stent extremities, in this case at 4 atm) and in the “final configuration” (i.e. when the nominal pressure value of 7 atm is reached) were computed and are reported in Table 4. The percentage errors in terms of stent inner diameter achieved at the end of the balloon pressure maintenance phase and the outer diameter at the end of the deflation phase (fully-expanded stent diameter) were also calculated and found to be 1.5% and 1.4%, respectively.

**Table 4:** Percentage error obtained by comparing the experimental and numerical stent diameters in the “post-dogboning configuration” and the “final configuration” of the free expansion test.

	Proximal Diameter		Central Diameter		Distal Diameter	
	Post-dogboning configuration	Final configuration	Post-dogboning configuration	Final configuration	Post-dogboning configuration	Final configuration
Errors [%]	2.5	1.0	4.7	1.7	1.8	0.4

#### 3.3.2 Stent tension testing

**Fig 8:**
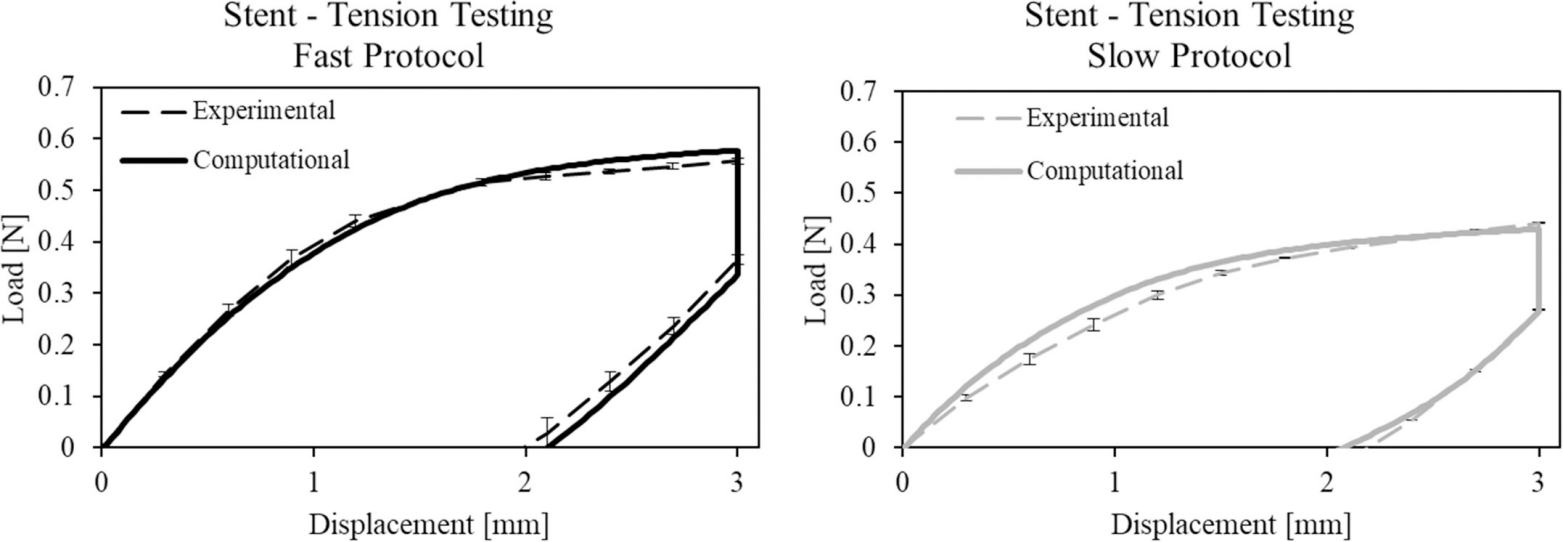
Comparison between experimental and computational force-displacement curves in tension testing of stents. Error bars indicate the maximum and minimum experimental value with respect to the mean value at the corresponding displacement.

Fig 8 shows the comparison between experimental and computational force-displacement curves related to uniaxial tension testing. The experimental data are shown plotting the mean load value with error bars to consider the variability between the minimum and maximum registered values.

The percentage errors obtained comparing the computational results with the experimental data are reported in Table 5.

**Table 5:** Percentage errors obtained comparing the experimental data with the computational results obtained with the calibration phase performed on the whole free-expanded stent.

Errors [%]	Maximum Force		Relaxation Force		Residual Plasticity	
	“Fast”	“Slow”	“Fast”	“Slow”	“Fast”	“Slow”
Whole stent	3.6	2.7	8.0	1.1	5.3	5.7

#### 3.3.3 Stent radial force testing

**Fig 9:**
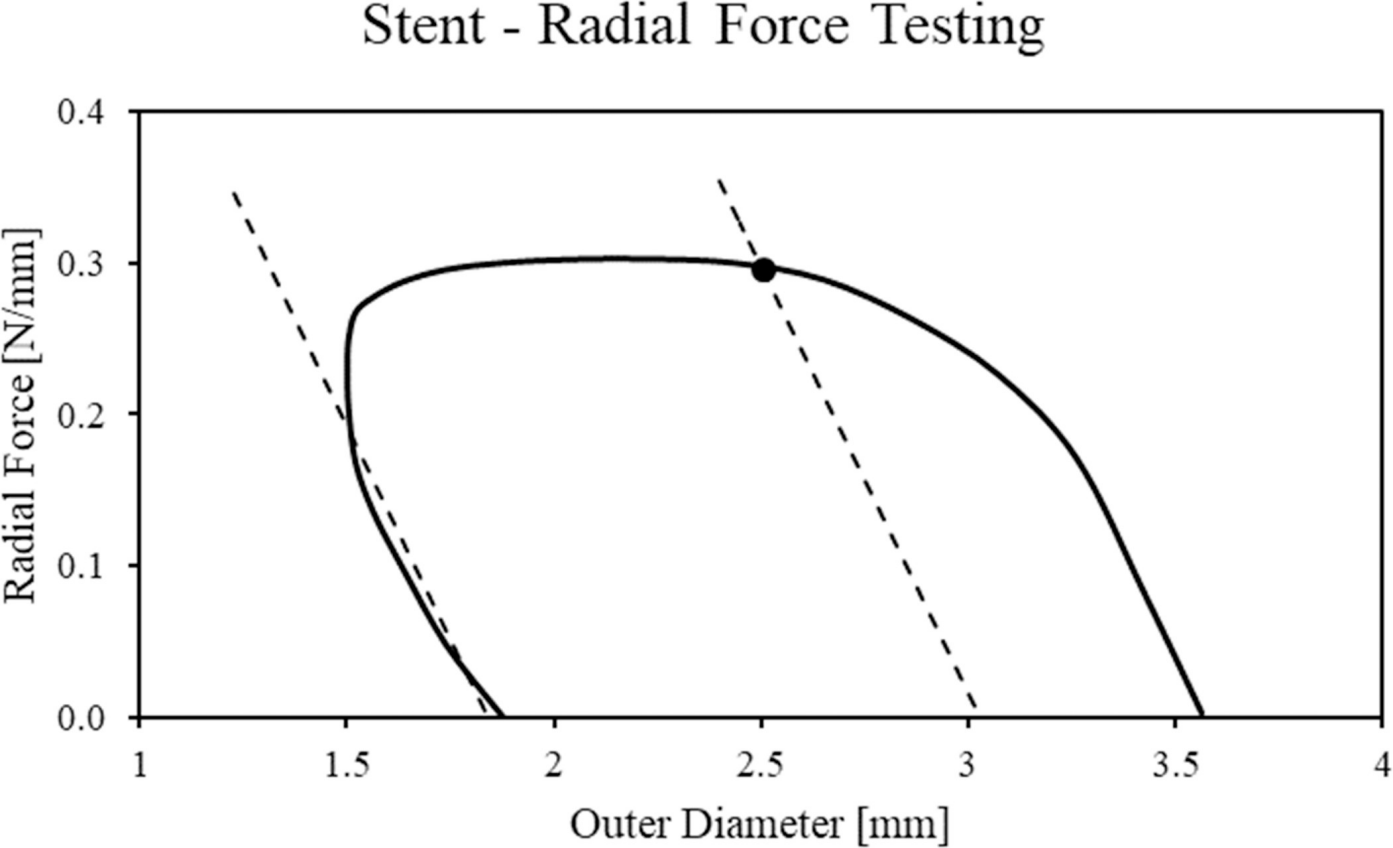
Numerical results of the recrimping simulation expressed in terms of diameter-radial force curve. A dashed line was used to indicate the slope of the curve during the unloading phase, which was used to find the intersection with the loading curve.

The computational results in terms of diameter-radial force curve are reported in Fig 9. In this curve, the first portion represents the mainly-elastic response of the device (constant slope), then the increase of plastic deformations is responsible for the plateau of the curve, followed by the final phase of crimping planes’ release. A value of radial strength equal to 0.29 N/mm was obtained, which is comparable with what was declared by the manufacturer of the device (0.22 N/mm).

## 4 Discussion

This work is placed in the context of the development of credible digital twins of cardiovascular devices. In particular, the topic of interest focuses on how to develop a reliable finite element model of a commercial bioresorbable polymeric stent, being available only a few samples of the device and no information on the material, which in this case is Tyrocore^TM^. This condition has to be faced when, for example, a clinician or a company wants to perform an independent study, for clinical planning or comparison purposes, respectively [17, 35–37]. A strategy, that involves the coupling of selected experimental and numerical activities, has been proposed to investigate and correctly simulate the mechanical response of the bioresorbable coronary delivery system.

Among the experiments, free expansion and tension testing on the whole stent were selected. Indeed, they can be easily replicated in any mechanical laboratory, do not require the use of dedicated testing machines (such as crimping machines), and are able to generate, at the local level, a stress state comparable with the one suffered by the stent in vivo, i.e. the bending of the struts. The choice of performing these tests according to different protocols (namely, “fast” and “slow”) was proven effective for the assessment of the visco-elastic and visco-plastic phenomena of polymers (such as the Tyrocore™), whose negligence might dramatically affect the simulation outcomes. The choice of performing the tests at two different paces derived from previous experience with the characterization of polymeric materials [6]. Since no material samples were available, and all tests involved whole devices, it was chosen to consider two testing velocities that could satisfy the following requirements: i) to appreciate and characterize the viscoelastic and viscoplastic behaviours; ii) to guarantee an accurate testing control; iii) to reduce the experimental and simulation time. As for the first requirement, the chosen velocities should be sufficiently different to properly appreciate all the rate-dependent phenomena; the second requirement was dictated by the characteristics of the testing apparatus, which cannot guarantee a proper control when very small displacements are applied in a reduced interval. As for the last point, regarding the experiments, it was decided to shorten the testing time for guaranteeing homogeneous conditions (e.g. temperature control); moreover, since the computational cost is strictly dependent on the loading rate, and the numerical step time must reflect the experiment duration, a long-lasting experiment is not recommended.

The truthful representation of the material phenomenology required the choice of a quite complex constitutive model, such as the PRF, characterized by several parameters [25]. Their calibration, due to the lack of literature data about the material mechanical behaviour and material samples to test, turned out to be quite difficult. For this reason, in this work, we proposed a strategy based on the exploitation of a simplified model, i.e. a portion of the stent (a single unit) that, if used with appropriate boundary and loading conditions, behaves as the whole device. Using the single unit, it was possible to run a high number of simulations. Many parameters combinations were inspected and the numerical results were compared to the data obtained from the experimental tests to select the set of data showing the best fitting. The choice of working with a reduced model was proven to be effective and functional for guaranteeing reliability but with a considerable save of computational time. Moreover, the proposed approach is potentially applicable to any device whose geometric structure is based on repetitive units.

The process was furtherly refined, considering also the residual stresses induced in the stent by the crimping phase during the delivery system production and the free expansion carried out prior to tension tests. It is worth noting that, for the specific case, the set of parameters found using the stress-free unit was suitable also for describing the behaviour of the stent after crimping. With different devices and materials, it might be necessary to perform a second procedure of parameter calibration using the single unit with residual stresses [22].

The performance of the generated model was evaluated on the whole stent by simulating free expansion and tension testing. Regarding free expansions, the digital twin of the device was able to correctly replicate the full-expanded configuration of the real stent. Once the dynamic phase of dogboning was completed, the computational diameter pressure curve reflected the behaviour experimentally observed (Fig 7), with a good match of the diameters measured at the end of the pressure-maintenance phase and after balloon deflation: the calculated errors are all below 5%. The aim of the study, developed in the framework of the European project Insilc, was indeed to have a model able to correctly reproduce the stent configuration at the end of the expansion (i.e. at the end of the deployment) and not the expansion dynamics, which is known to be extremely variable and difficult to replicate in numerical simulations [16].

Satisfactory results also emerged from the comparison with axial tension testing, in which it can be clearly seen that the model correctly described the changes in mechanical performance due to different load speeds: the calculated errors are all below 8%.

All the experimental and computational data used for model development and validation (from Figs 5–9) are collected in the S1 File.

The main limitation of the work is the reduced number of devices, which imposed a strict selection of experimental tests and protocols, considering also the necessity of investing some samples to increase the awareness of the experimental variability [38]. Indeed, with more samples, it would have been possible to carry out further experimental tests, such as crush or radial force testing, and investigate additional speeds from those used in this work. Clearly, this would have allowed an extensive validation of the developed model and a more complete assessment of the realism of the visco-elastic and visco-plastic performance.

## 5 Conclusions

The possibility of using simulations in the cardiovascular field, for improving devices and procedures, is strictly related to their capability of correctly describing the reality of interest. In particular, for setting up the model of a device it is necessary to have specific information, for correctly defining the geometry and material properties, and to assess the in silico model with in vitro or in vivo results. Numerical studies carried out independently from the manufacturer are very useful to increase knowledge and investigate the limits and potentialities of commercial devices: this is even more true when dealing with new materials with complex behaviour, strongly dependent on loading and environmental conditions, as for BVS. However, these studies are hampered by the difficulty of having sufficient data to build reliable models. In this work, a pathway has been proposed, combining few experimental tests with several simulations of increasing complexities, for obtaining a reliable model for bioresorbable coronary stent deployment simulation, only a few commercial delivery systems being available. The outlined pathway allowed obtaining a numerical model able to predict with an error lower than 10% the final configuration of the stent after free-expansion and tension testing. It is possible to conclude that if the context of use is well defined, even when few data are available, it is possible to define a strategy that supports the appropriate use of the available resources to build a suitable model and assess its credibility.

## Supporting information

S1 File Supporting information file containing all the experimental and computational data used for model development and validation. (XLSX)

## Abbreviations

BMS: Bare metal stent
BVS: Bioresorbable vascular scaffold
CVD: Cardiovascular disease
DES: Drug-eluting stent
FE: Finite element
PRF: Parallel rheological framework
PTA: Percutaneous transluminal angioplasty
SR: Stiffness Ratio
${\bar{\varepsilon }}^{cr}$: equivalent creep strain
${\dot{\bar{\varepsilon }}}^{cr}$: equivalent creep strain rate
${\dot{\varepsilon }}_{pl}$: equivalent plastic strain rate
σ_y_: yield stress
*σ*_*y*0_: Static yield stress
A: material parameter defining viscous behaviour
*C*_10_: material parameter defining hyper-elastic behaviour
*D*_1_: material parameter defining hyper-elastic behaviour
e: material parameter defining plastic behaviour
${\bar{I}}_{1}$: first deviatoric strain invariant
J^el^: elastic volume ratio
m: material parameter defining viscous behaviour
mult: material parameter defining plastic behaviour
n: material parameter defining viscous behaviour
$\tilde{q}$: equivalent deviatoric Kirchhoff stress
